# FGF23 and Cell Stress in SaOS-2 Cells—A Model Reflecting X-Linked Hypophosphatemia Dynamics

**DOI:** 10.3390/cells13181515

**Published:** 2024-09-10

**Authors:** Lisanne Brueck, Sascha Roocke, Veronika Matschke, Annette Richter-Unruh, Katrin Marcus-Alic, Carsten Theiss, Sarah Stahlke

**Affiliations:** 1 Department of Cytology, Institute of Anatomy, Ruhr-University Bochum, D-44801 Bochum, Germany; lisanne.brueck@ruhr-uni-bochum.de (L.B.);; 2 The Medical Proteome Center, Ruhr-University Bochum, D-44801 Bochum, Germany; 3 International Graduate School of Neuroscience (IGSN), Ruhr-University Bochum, D-44801 Bochum, Germany; 4 Clinic for Children and Adolescents, Pediatric Endocrinology, St. Josefs-Hospital, D-44791 Bochum, Germany

**Keywords:** FGF23, ER stress, mitochondrial stress, TEM, Western Blot, XLH, X-linked hypophosphatemic rickets, cell stress, rare diseases

## Abstract

Our study investigates the impact of FGF23 overexpression on SaOS-2 cells to elucidate its role in cellular stress and morphology, contributing to the understanding of skeletal pathologies like X-linked hypophosphatemia (XLH). Using transmission electron microscopy and protein analysis (Western blot), we analyzed the rough endoplasmic reticulum (rER) and mitochondria in SaOS-2 cells with FGF23 overexpression compared to controls. We found significant morphological changes, including enlarged and elongated rER and mitochondria, with increased contact zones, suggesting enhanced interaction and adaptation to elevated protein synthesis and secretion demands. Additionally, we observed higher apoptosis rates of the cells after 24–72 h in vitro and upregulated proteins associated with ER stress and apoptosis, such as CHOP, XBP1 (spliced and unspliced), GRP94, eIF2α, and BAX. These findings indicate a robust activation of the unfolded protein response (UPR) and apoptotic pathways due to FGF23 overexpression. Our results highlight the critical role of ER and mitochondrial interactions in cellular stress responses and provide new insights into the mechanistic link between FGF23 signaling and cellular homeostasis. In conclusion, our study underscores the importance of analyzing UPR-related pathways in the development of therapeutic strategies for skeletal and systemic diseases and contributes to a broader understanding of diseases like XLH.

## 1. Introduction

Skeletal diseases are a major global health problem, affecting individuals across all demographic groups and posing significant challenges to healthcare systems in terms of both direct medical costs and indirect costs associated with disability and lost productivity [1]. These diseases, which can be attributed to various factors such as genetic mutations, metabolic disorders, and tumor processes, underscore the complexity of maintaining skeletal health. Among these pathologies, X-linked hypophosphatemia (XLH) stands out as a paradigmatic example, characterized by aberrant phosphate metabolism and skeletal deformities [2].

Dysregulation of fibroblast growth factor (FGF) 23 signaling is central to the pathogenesis of XLH. FGF23, a member of the fibroblast growth factor (FGF) superfamily, consists of a class of 22 polypeptides with critical roles in embryonic and postnatal development, as well as cell cycle regulation [3]. It exerts pivotal control over phosphate and vitamin D metabolism primarily within bone tissue [4]. Dysfunction in FGF23 signaling pathways, whether due to genetic abnormalities or other factors, can lead to systemic disorders such as XLH, the most common form of hereditary rickets, characterized by skeletal abnormalities and metabolic perturbations [5,6,7], thereby emphasizing the critical role of FGF23 in skeletal homeostasis [5].

**Figure 1:**
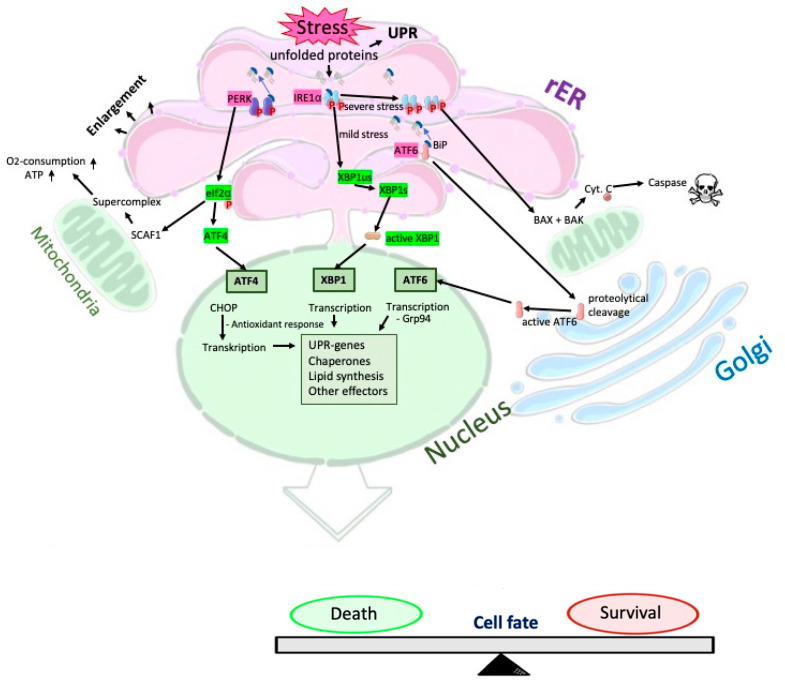
Overview of subcellular ER regulatory processes and reaction circuits triggered by stress.

Emerging research has highlighted the significance of endoplasmic reticulum (ER) stress in the pathogenesis of various diseases, including atherosclerosis, diabetes, and obesity [8]. The ER, responsible for protein synthesis and folding, plays a central role in maintaining cellular homeostasis. Under conditions of metabolic dysregulation or pathological stress, the ER can undergo stress-induced dysfunction, triggering the unfolded protein response (UPR) and potentially leading to cellular dysfunction or apoptosis, as summarized in Figure 1.

The UPR augments ER size to enhance protein folding capacity, initiated by IRE1α, PERK, and ATF6 [9]. Unresolved ER stress can trigger IRE1α-induced apoptosis via hyperactivation and homo-oligomerization, involving mitochondrial protective proteins and proapoptotic factors like Bcl-2-like protein 4 (BAX) and Bcl-2-like protein 7 (BAK), leading to controlled cell death [10]. ER stress-mediated apoptosis is implicated in numerous chronic diseases [11]. If cells survive ER stress, antioxidant responses and ER chaperones are upregulated [10]. Upregulated calcium-binding proteins, including calreticulin (CALR), Grp94, 78 kDa glucose-regulated protein (Grp78), and ER protein 72 (Erp72), are part of the ATF6 signaling cascade [12,13,14].

Mitochondrial dysfunction due to metabolic changes induces mitochondrial stress as part of the stress response [15]. In addition, mitofusin 2 (MFN2) regulates ER-mitochondria contact sites, affecting ER shape and inter-organelle interactions [16,17].

Understanding the intricate interplay between FGF23 dysregulation, ER stress, and cellular responses is crucial for elucidating the molecular mechanisms underlying skeletal pathologies such as XLH. Despite its rarity, XLH serves as a model system for exploring fundamental biological processes underlying bone diseases, offering insights that may have broader implications for skeletal health.

The present study investigates the impact of FGF23 overexpression on cellular structures and stress responses using SaOS-2 cells as a model system, revealing that FGF23 alters the structure of mitochondria and the rough ER (rER) and their interactions. SaOS-2 cells, a well-established human osteosarcoma cell line, are used in scientific research for the investigation of bone biology and associated processes. These cells closely mimic the functional properties of osteoblasts, the primary cells responsible for bone formation [18]. Additionally, SaOS-2 cells have been used to assess the cytotoxicity of various compounds and their effects on cell proliferation, apoptosis, and the unfold protein response (UPR) [19,20]. By elucidating these mechanisms, this work contributes to the broader effort to improve diagnostic strategies and develop targeted interventions aimed at preserving bone health and enhancing patient outcomes.

## 2. Materials and Methods

### 2.1. Cell Culture

#### 2.1.1. SaOS-2 Cells

**Figure 2:**
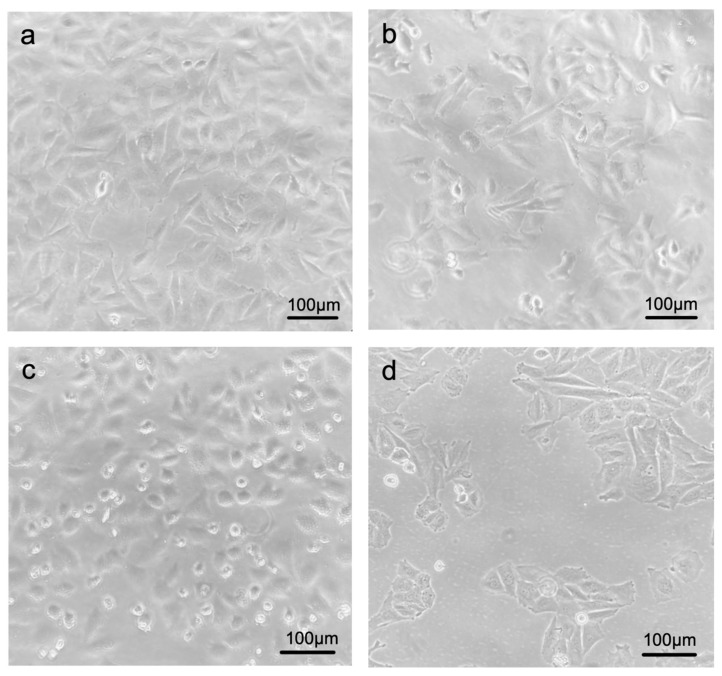
Light microscopic phase-contrast images of SaOS-2 cells. SaOS-2 cells with empty vector (**a**,**b**) and FGF23 overexpression (**c**,**d**) under the phase contrast microscope in different confluences.

Human SaOS-2 cells (#89050205, Sigma-Aldrich, Taufkirchen, Germany) were cultivated in T75 cell culture flasks in growth medium containing Minimal Essential Medium Eagle (M7278, Sigma-Aldrich, Taufkirchen, Germany) supplemented with 10% FBS (F2442, Sigma-Aldrich, Taufkirchen, Germany), 1% penicillin/streptomycin (PS-B, Capricorn-Scientific, Ebsdorfergrund, Germany), 1% Glutamax (#35050-061; Thermo Fisher Scientific, Darmstadt, Germany) and 0.55 ug/mL puromycin (ant-pr-1, Thermo Fisher Scientific, Darmstadt, Germany) in a humidified incubator at 37 °C and 5% CO_2_. The growth medium was refreshed every 3 days. When approx. 80–90% confluence was reached, the cells were either taken into the experiment (see Figure 2) or split to be cultured as required. For splitting, the cells were first digested with 2 mL trypsin with EDTA (#T4049, Sigma-Aldrich, Taufkirchen, Germany) for about 5 min; the successful digestion was verified under the microscope. Subsequently, 4 mL of medium (see above) were used, and after a 5 min centrifugation at 1000 rpm (Beckmann Coulter, Krefeld, Germany), 1:4 splitting was performed to obtain 4 new culture flasks from one T 75 culture flask (#833911002, Sarstedt, Nümbrecht, Germany).

#### 2.1.2. Generation of FGF23 Overexpressing and Control Empty Vector Cells

The cells were generated by the Medical Proteome Center (MPC) in cooperation with the Department of Molecular GI-Oncology (MGO, both Ruhr-University Bochum, D-44801 Bochum, Germany). In brief, stable overexpression of FGF23 in SaOS-2 cells was induced by lentiviral transduction. A FGF23-6 His coding gene (derived from #RC210127, Origene, Herford, Germany) was induced into the lentiviral plasmid pLJM (#19319, Addgene, Teddington, UK), while non-coding pLJM was used as an empty vector control (EV). The pLJM vectors were mixed with the lentiviral envelope plasmid pCMV-VSV-G (#8454, Addgene, Teddington, UK), the packaging plasmid gag/pol (Addgene #14887, Teddington, UK) (ratio 2:1:2), HBS buffer, and 125 mM CaCl2 solution and added to the medium of lentivirally infected packaging cells (Human Embryonic Kidney cells, HEK). After an incubation of 24 h, the medium was replaced, followed by a second incubation of 24 h. Finally, the medium was removed from the packaging cells, sterile filtered (0.45 µm), and added to SaOS-2 cells at 50% confluence for another overnight incubation. To select transduced cells, 0.5 µg/µL pyromycin was added to the culture medium. Initial experiments showed no significant difference between wild-type and empty vector cells.

#### 2.1.3. Human-Embryonic-Kidney (HEK) Cells

Three days after the last medium refreshment, the hole medium, from now on referred to as “supernatant” from SaOS-2 cells, was transferred to HEK cells. After two hours of incubation, RNA from the HEK cells was isolated and subsequently measured by real-time PCR (see below, “Quantitative reverse transcription polymerase chain reaction (qPCR)”). On the one hand, the supernatants of SaOS-2 cells overexpressing FGF23 were used for the experiment, while the supernatants of empty vector SaOS-2 cells were used in the control groups. HEK cells were cultured in T75 cell culture flasks (#833911002, Sarstedt, Nümbrecht, Germany) in growth medium consisting of Dulbecco’s Minimal Essential Medium Eagle (#M4655, Sigma-Aldrich, Taufkirchen, Germany) supplemented with 10% FCS (#FBS-11A, Capricorn-Scientific, Ebsdorfergrund, Germany), 1% penicillin/streptomycin (PS-B, Capricorn-Scientific, Ebsdorfergrund, Germany), and 1% sodium pyruvate (#NPY-B, Capricorn-Scientific, Ebsdorfergrund, Germany) in a humidified incubator at 37 °C and 5% CO_2_. Growth medium was refreshed every 3 days.

### 2.2. Transmission Electron Microscopy (TEM)

Confluent SaOS-2 cells were washed with PBS and fixed for one hour with 2% glutaraldehyde (#G5882, Sigma-Aldrich, Taufkirchen, Germany) in 0.2 mol/L cacodylate buffer (#AGR1503, agarscientific, Essex, UK) (pH 7.2) at room temperature. This was followed by incubation with 2% osmium tetroxide (#0972B-6, Polyscience Inc., Hirschberg, Germany) for 90 min. The cells were then collected using a cell scraper and fixed in agar blocks. Samples were then dehydrated in an ascending ethanol series, starting with 50% ethanol, followed by incubation in 70% ethanol, 1% uranyl acetate (#21447, Polyscience Inc., Hirschberg, Germanyand 1% phosphotungstic acid (#455970, Sigma-Aldrich, Taufkirchen, Germany) overnight at 4 °C. Dehydration continued the next day with an ascending ethanol series (80–100%). The samples were carefully transferred into epoxy resin (consisting of glycidether (#21045.02, Serva, Heidelberg Germany), methylnadic anhydride (#29452.02, Serva, Heidelberg, Germany), 2-dodecenyl succinic anhydride (#20755.01, Serva, Heidelberg Germany), and 2,4,6-tris (dimethylaminomethyl)phenol (#36975.01, Serva Heidelberg, Germany) and incubated in propylene oxide (#807027, Sigma-Aldrich, Taufkirchen, Germany). After polymerization, ultrathin slices (50–70 nm) were cut using an Ultracut E Reichert-Jung (Leica Microsystems GmbH, Wetzlar, Germany) with a DiATOME histo diamond knife (45°, 6 mm, MX559; Diatome AG, Switzerland). Images were captured with a Zeiss Leo 910 (Zeiss, Oberkochen, Germany) equipped with a digital CCD camera and analyzed using ImageJ 1.51 s (National Institutes of Health, USA).

### 2.3. Evaluation and Measurement of the TEM Images

To get a good overall impression, images of SaOS-2 cells were taken from different sections, and various parameters were considered for morphological characterization. In particular, the expansion of the cell organelles was quantified. The (1) area in µm^2^ with a spatial calibration of the corresponding scale bar in each picture. (2) FeretMin as the smallest possible distance between two parallel tangents on the sides of the rER at any angle. (3) aspect ratio defined as:

$$\frac{major\;axis}{minor\;axis}$$

(4) The length of the outside boundary of the selection as perimeter. (5) circularity, calculated by:

$$\frac{4\pi \;\times \;Area}{Perimeter^{2}}$$

In addition, (6) roundness as:

$$\frac{4\;\times \;Area}{\pi \;\times \;Major\;Axis^{2}}$$

were considered. A total of 698 ER structures and 787 mitochondria were selected from 188 images using ImageJ 1.51 s software (National Institutes of Health, USA) and measured in a blinded manner. An unpaired *t*-test was performed for significance testing between the two cell lines, and values with *p* < 0.05 were considered significant. 

### 2.4. Measurement of ER-Mitochondria Contacts

**Figure 3:**
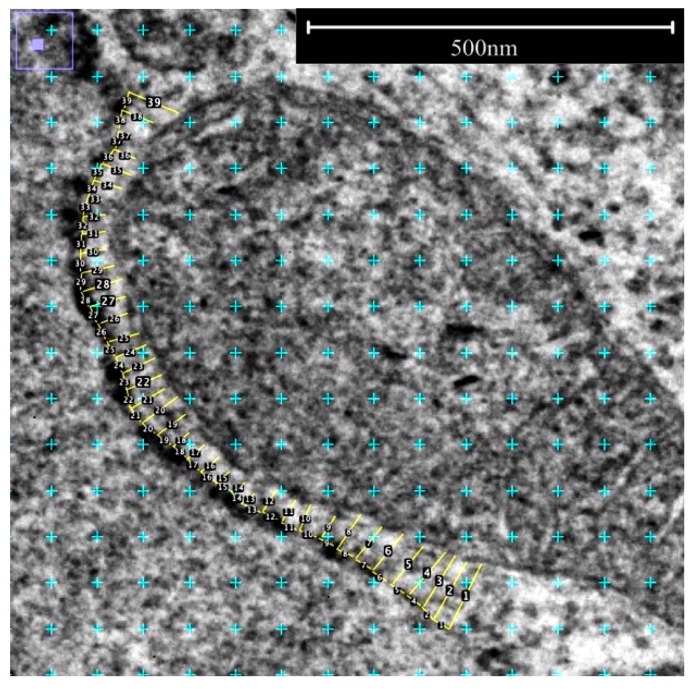
Exemplary measurement of the contact zones between rER and mitochondria.

To assess ER-mitochondria contacts, the distance from the ER to the mitochondria was measured with the “length tool” in ImageJ (Figure 3, yellow lines, numbered). First, the outline of the cell organelle was marked and circled along the contact zone. Measurements were taken at intervals of 25 nm where the length (distance) between the two structures was determined. All measurements were carried out blinded. Although the contact zones have been measured over a large part of their extent (see Figure 3), only contact zones up to 25 nm were used for the evaluation, as these are relevant as described by Csordás et al. 2006 [21]. In total, 44 contact zones with 1025 measurements were performed in FGF23 overexpressing cells (FGF23) and 28 contact zones with 442 measurements in the control (EV) cells. An illustrative example of these measurements is provided in Figure 3 for better visualization.

### 2.5. Western Blots

For Western blot analysis, confluent SaOS-2 cells were detached from the culture flasks using EDTA-Trypsin (#T4049, Sigma-Aldrich, Taufkirchen, Germany), and subsequently proteins were extracted using cell lysis (CL) (#FNN0011, ThermoFisher Scientific, Darmstadt, Germany) buffer containing additionally protease inhibitors (#11873580001, Roche, Mannheim, Germany). All procedures were conducted on ice to preserve protein integrity. The solution was gently shaken for 30 min at 4 °C, followed by centrifugation at 12,000 rpm for 10 min, and the resulting supernatant was collected for further western blot analysis. Protein concentrations were determined using the Pierce™ BCA Protein Assay Kit (# 23225, Thermo Fisher Scientific, Darmstadt, Germany). Prior to protein separation, samples were heated at 90 °C for five minutes. A 4× Laemmli sample buffer (#161-0747, BioRad, Feldkirchen, Germany) was added to the samples. Proteins were separated by sodium dodecyl sulfate-polyacrylamide gel electrophoresis at 100 V, followed by transfer onto PVDF membranes using the Trans-Blot^®^ Turbo™ Transfer System (#1704150, BioRad. Feldkirchen, Germany) according to the manufacturer’s protocol. Membranes were then blocked for at least one hour using either 1× phosphate-buffered saline (PBS) with 1% RotiBlock (#A151 Roth, Karlsruhe, Germany) or 5% BSA (#fraction V; Sigma-Aldrich, Taufkirchen, Germany) or 5% skim milk, depending on the primary antibody used. Primary antibodies (Table 1) were incubated overnight at 4 °C, followed by incubation with secondary antibodies (Table 1) for 2 h at room temperature. Protein bands were visualized using Western Blotting Luminol Reagent (#sc-2048, Santa Cruz Biotechnology, Heidelberg, Germany). For semiquantitative analysis, the band intensities of the proteins of interest were normalized to the loading control. All values were then further normalized to the mean of the normalized empty vector SaOS-2 cell signals. Western blot analysis was performed on tissue samples from at least three FGF23 overexpressing and three empty vector samples. 

### 2.6. Immunofluorescence

For staining, coverslips were placed in a Petri dish containing Minimal Essential Medium (see “Cell culture–Section 2.1”) and incubated in a humidified incubator at 37 °C to equilibrate. Subsequently, previously trypsinized SaOS-2 cells from culture were added to the coverslips and cultivated under the same conditions for 1 to 3 days to measure apoptosis. Following incubation, the medium was discarded, and coverslips were washed with PBS before fixation with 4% PFA. Staining was carried out according to a standard protocol, with 0.1% PBS-Triton for permeabilization and triple washes in PBS between all steps. Primary antibodies (phalloidin 1:50, #ab176757, abcam; cleaved caspase 3 1:100, #9664, Cell signalling, Leiden, Netherlands) were incubated overnight at 4 °C, followed by incubation with secondary antibodies (anti-rabbit IgG (H+L) highly cross-adsorbed secondary antibody, 1:200, #A-21206, Thermo Fisher Scientific, Darmstadt, Germany) at room temperature for 2 h. Nuclear staining was performed using 1 mg/mL DAPI for 30 min at room temperature. After mounting, samples were allowed to dry, and images were captured using a Keyence microscope (BZ-X800, Keyence Corporation, Osaka, Japan).

Apoptotic cells could be identified by cleaved caspase 3. To determine the apoptosis rate, the percentage of apoptotic cells (caspase 3 positive) in the total number of cells (DAPI positive) was calculated and analyzed in a blinded manner. 

### 2.7. Quantitative Reverse Transcription Polymerase Chain Reaction (qPCR) 

For qRT-PCR analysis, confluent SaOS-2 cells were detached from the culture flasks using EDTA-Trypsin, followed by centrifugation at 1200 rpm for 5 min to collect the cell pellets on ice. RNA isolation from the cell pellet was carried out using the NucleoSpin RNA kit, followed by reverse transcription into complementary deoxyribonucleic acid (cDNA) using the GoScript™ Reverse Transcription Mix, Oligo (dT) (#A2791, Promega, Madison, WI, USA), as per the manufacturer’s instructions.

The qRT-PCR was performed with a CFX96 Real-Time PCR Detection System (Bio-Rad, Hercules, CA, USA), employing GoTag qPCR Master Mix (Cat# A6001, Promega, Walldorf, Germany), 100 ng cDNA per reaction, and the appropriate primers (Table 2). The qRT-PCR analyses were performed in at least quadruplicate, using GAPDH and actin as housekeeping genes.

The 2^−ΔΔct^ method was applied [22], using the mean ct-value normalized to the housekeeping genes GAPDH and actin, as well as the empty vector cells as reference.

### 2.8. Statistical Evaluations

Statistical data analysis was performed with Prism 7.0 (GraphPad Inc., La Jolla, CA, USA). Data are represented as means of independent experiments ± standard deviation (SD) as indicated. Significance testing was conducted using Student’s *t*-test or two-way ANOVA with a Sidak post-hoc test. Results with *p* < 0.05 were considered statistically significant. The ROUT method with a Q-value of 1% was employed to identify outliers.

## 3. Results

### 3.1. FGF23 Influences the Structure of Mitochondria and the rER and Alters Their Interactions

#### 3.1.1. Rough Endoplasmic Reticulum

**Figure 4:**
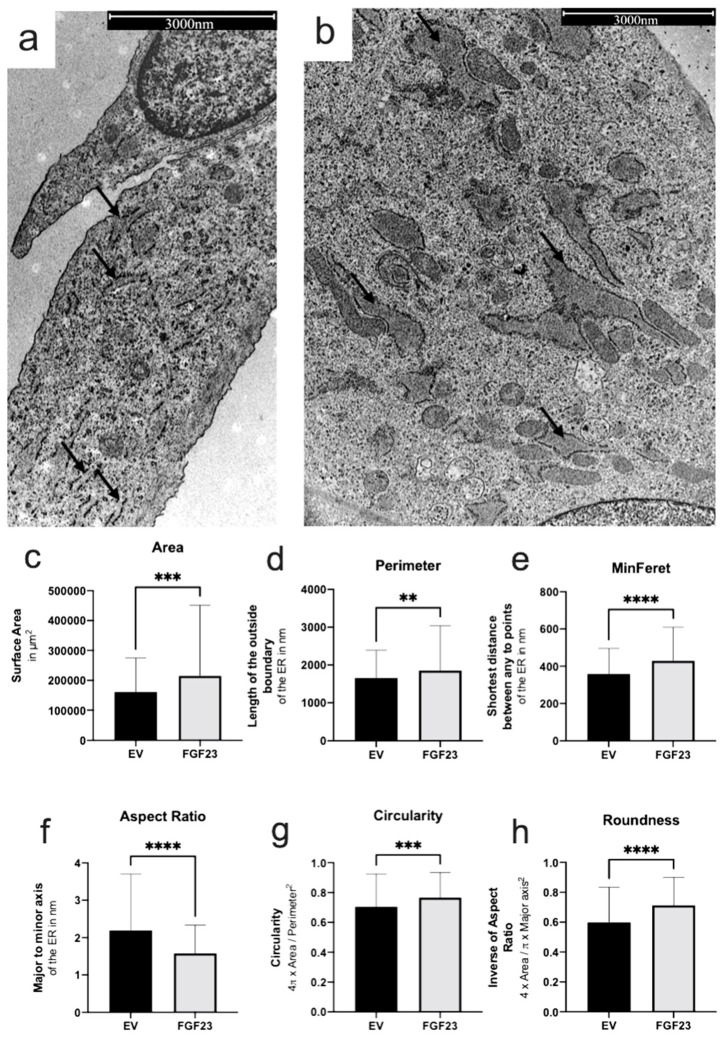
TEM images of the rERs and statistical evaluation for n = 698 analyzed ER-strucutres. Electron microscopy images of SaOS-2 cells with empty vector (EV; control; (**a**)) and with FGF23 overexpression (FGF23; (**b**)). Arrows: rER (exemplary). Statistical analyses of rER area (**c**), perimeter (**d**), smallest possible distance between two parallel tangents on the sides of the rER at any angle (FeretMin; (**e**)), the aspect ratio (**f**), similarity to a perfect circle (Circularity; (**g**)) and the degree of roundness (**h**) were performed. Statistically significant changes are indicated as ** *p* < 0.01, *** *p* < 0.001, and **** *p* < 0.0001.

The cellular morphology of the rER in SaOS-2 was examined using transmission electron microscope (TEM) images (Figure 4a,b). Control cells were transfected with empty vectors (EV), while the experimental group overexpressed FGF23. In the analysis of the rER, the area (Figure 4c) was significantly increased in FGF23 overexpressing cells compared to control cells (mean surface: 160.842 µm^2^ vs. 215.130 µm^2^, *p* = 0.0002). Similarly, the rER perimeter was significantly larger in FGF23 cells (1858 µm) compared to EV (1655 µm, *p* = 0.0187; Figure 4d). The mean shortest distance (FeretMin) between any two points of the rER was also significantly greater in FGF23 cells (429.8 µm) than in EV (358.9 µm, *p* < 0.0001; Figure 4e). Additionally, the aspect ratio indicated a more elongated rER shape in FGF23-overexpressing cells (*p* < 0.0001; Figure 4f). Circularity was significantly higher in FGF23 cells compared to EV (*p* = 0.0001; Figure 4g), indicating a rounder shape in the former. Similarly, the roundness (Figure 4h) was significantly higher in FGF23 cells compared to control cells (EV; *p* < 0.0001). These findings suggest profound morphological alterations in the rER of FGF23-overexpressing cells, as reflected in significant changes in diameter, circumference, and overall shape.

#### 3.1.2. Mitochondria

**Figure 5:**
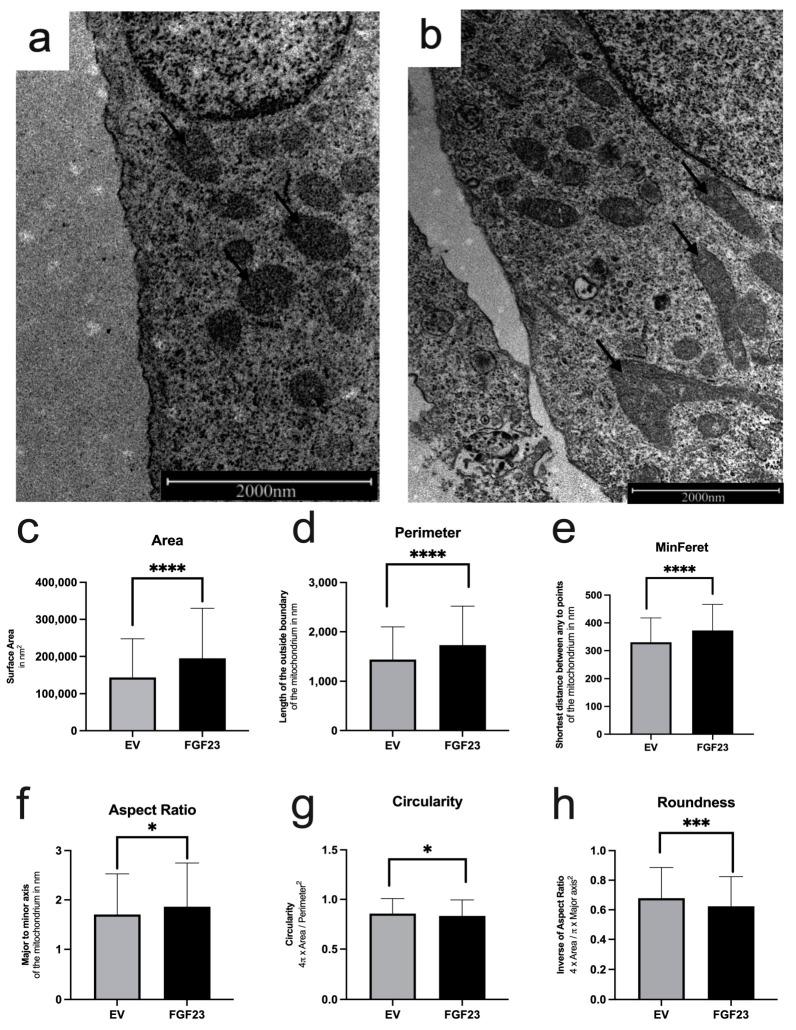
TEM images of the analyzed mitochondria (n = 787) and statistical evaluation. Electron microscopy images of SaOS-2 cells with empty vector (EV; control; (**a**)) and with FGF23 overexpression (FGF23; (**b**)). Arrows: Mitochondria (exemplary). Statistical analyses of mitochondrial area (**c**), perimeter (**d**), smallest possible distance between two parallel tangents on the sides of the rER at any angle (FeretMin; (**e**)), the aspect ratio (**f**), similarity to a perfect circle (Circularity; (**g**)), and the degree of roundness (**h**) were performed. (* *p* < 0.05, *** *p* < 0.001, and **** *p* < 0.0001).

The mitochondria in both control cells with empty vectors (EV) and FGF23 overexpressing cells (FGF23) were examined using transmission electron microscopy (TEM) (Figure 5a,b). In the analysis of the mitochondrial area (Figure 5c), a significant increase was observed in FGF23 overexpressing cells compared to control cells with empty vectors (EV; mean surface: 1444.049 µm^2^ vs. 195.803 µm^2^; *p* < 0.0001). Similar significant differences were found in the measured perimeter (Figure 5d) of mitochondria, with EV measuring 1.440 µm and FGF23 measuring 1.730 µm (*p* < 0.0001). The mean shortest distance between any two points (FeretMin, Figure 5e) also showed a significant increase in FGF23 overexpressing cells compared to EV (330.1 µm vs. 372.9 µm, *p* < 0.0001). The aspect ratio (Figure 5f) was significantly higher (*p* = 0.01362) in FGF23 compared to EV, indicating a more elongated shape. Additionally, the circularity was significantly lower in FGF23 compared to EV (*p* = 0.0471), indicating a less round shape (Figure 5g). This trend was consistent in the roundness measurements as circularity was corrected by aspect ratio as well (*p* = 0.0004) (Figure 5h).

Despite the larger size of mitochondria in cells with increased FGF23, they exhibited a less rounded shape, suggesting an uneven deformation accompanied by an overall increase in size. This observation indicates a notable change in mitochondrial structure under the influence of FGF23, which is consistent with the alterations observed in the rough endoplasmic reticulum (rER) morphology.

#### 3.1.3. rER-Mitochondria Contact Zones

**Figure 6:**
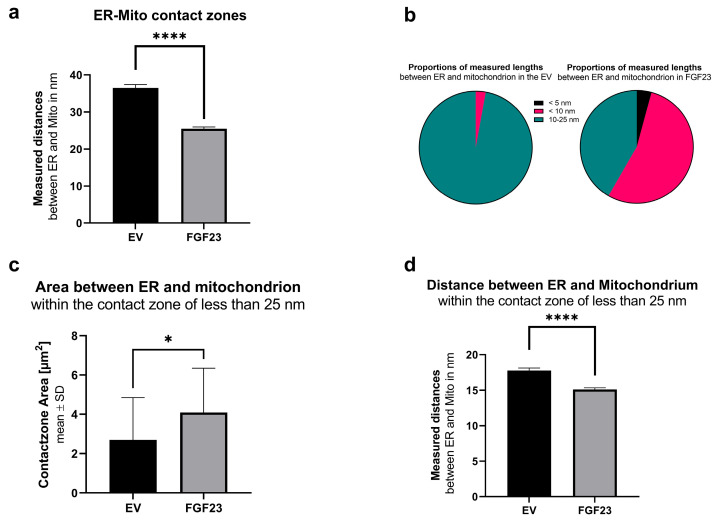
Statistical evaluation of contact zones between rER and mitochondria. (**a**) Measured lengths of all distances within the contact zones showed significant closer contact to each other under the influence of FGF23. (**b**) Proportions of measured lengths (<5 nm, <10 nm, and 10–25 nm) between ER and mitochondrion in the EV (left; N = 28 contact zones; n = 442 measurements) and FGF23 (right; N = 44 contact zones; n = 1025 measurements). (**c**) The measured area within the contact zone of less than 25 nm is enlarged in FGF23 compared to EV. (**d**) The distance between ER and mitochondria is shorter under FGF23 influence. (* *p* < 0.05 and **** *p* < 0.0001).

Contact zones between mitochondria and the rER were analyzed, as described before (Figure 6). FGF23 induces a significant reduction in the distance between cell organelles, frequently resulting in inter-organelle distances of less than five nanometers. (Figure 6b).

The measured distances between the structures (Figure 6a) were found to be significant (*p* < 0.0001) different, with an average distance of 36.52 nm for EV compared to 25.49 nm in FGF23 cells. Under control conditions (EV), no contact zones shorter than 5 nm could be detected, and only 2.857% of all measured contacts are shorter than 10 nm. In contrast to this, FGF23 overexpression in the cell leads to 4.218% of contacts <5 nm, 54.13% < 10 nm, and 41.652% between 10 and 25 nm. In consistent with these results, FGF23 overexpression also revealed that the contact zone is significantly (*p* = 0.0197) larger in the complete range below 25 nm (Figure 6c), and rER and mitochondria are significantly (*p* < 0.0001) closer (Figure 6d) in this range compared to the EV cell.

The analysis indicates that under the influence of FGF23, the dynamic cell organelles, rER and mitochondria, come closer to each other, as evidenced by more frequent, closer, and larger contact.

### 3.2. Impact of FGF23 Overexpression on Morphology, Stress, and Apoptosis in SaOS-2 Cells

#### 3.2.1. Morphology and Apoptosis

**Figure 7:**
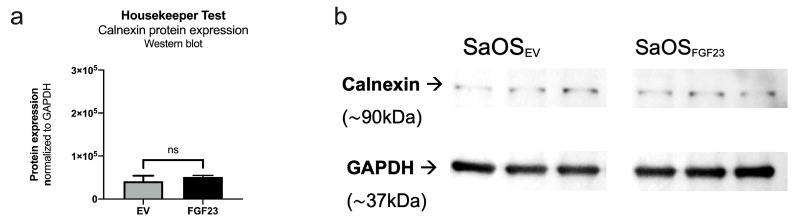
Housekeeper test. Western blots (**b**) for Housekeepers Calnexin and GAPDH showing no significant (ns) differences in expression comparing SaOS-2 cells transfected with and empty vector (SaOSEV) with SaOS-2 cells overexpressing FGF23 (SaOS_FGF23_). Quantification (**a**) of the protein expression [n = 3].

The morphological examination of SaOS-2 cells under the light microscope shows a consistently healthy appearance of the living cells both in control SaOS-2 cells transfected with an empty vector as well as in the cells overexpressing FGF23 (Figure 2). Furthermore, there is no influence of FGF23 overexpression on the expression levels of the “classical” housekeeping genes calnexin and GAPDH at the protein level (Figure 7).

An increased number of dead cells is clearly visible in the SaOS-2 cell culture flasks with FGF23 overexpression without the use of high magnification (Figure 2).

**Figure 8:**
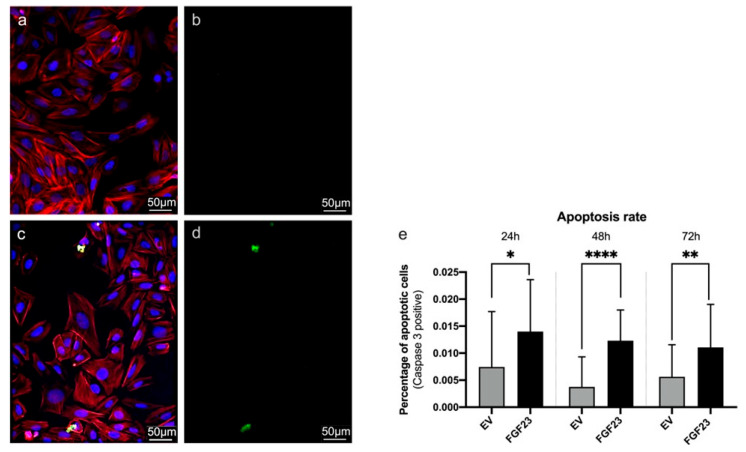
Flourescence microscopic determination of apoptosis. Shown are exemplary images of cells with empty vector (**a**,**b**) and with FGF23 overexpression (**c**,**d**). Fluorescence microscopy images of SaOS-2 cells with DAPI staining of the cell nuclei (blue), filamentous actin (red) and cleaved-caspase 3 (green). (**a**,**c**) overlay of all stained structures, (**b**,**d**) show only the apoptotic cells (staining in green (cleaved-caspase 3)). (**e**) Statistical evaluation of apoptosis. The percentage of apoptotic cells was determined after 24, 48, and 72 h. At all observed time points, cells overexpressing FGF23 have significantly more cells positive for cleaved caspase 3. (* *p* < 0.05, ** *p* < 0.01, and **** *p* < 0.0001) [n = 4 for every time point, with each trial having 3 slides containing 30–300 cells].

Immunohistochemical analysis of the apoptotic marker Caspase 3 over a period of 3 days (24 h, 48 h, and 72 h) in culture reveals a significantly increased apoptosis rate in FGF23-overexpressing cells compared to empty vector (EV) control cells at all three investigated time points (*p* = 0.0267 (24 h), *p* < 0.0001 (48 h), *p* = 0.0100 (72 h); Figure 8). The effect is most pronounced after 48 h in culture. The overall low cell death rate in all cultures (less than 0.1 percent) indicates a healthy culture. In both cultures analyzed, cell death is lowest at 24 h (EV: 0.0074 ± 0.0103; FGF23 0.0140 ± 0.0096); while it fluctuates in the control, it decreases over time in the FGF23 overexpressing culture.

This observation suggests that while FGF23 overexpression does not visibly alter the morphology of SaOS-2 cells or affect the expression of housekeeping genes, it does lead to a heightened apoptotic response, indicating a potential role of FGF23 in regulating apoptosis pathways in these cells.

#### 3.2.2. FGF23 Expression and Secretion

**Figure 9:**
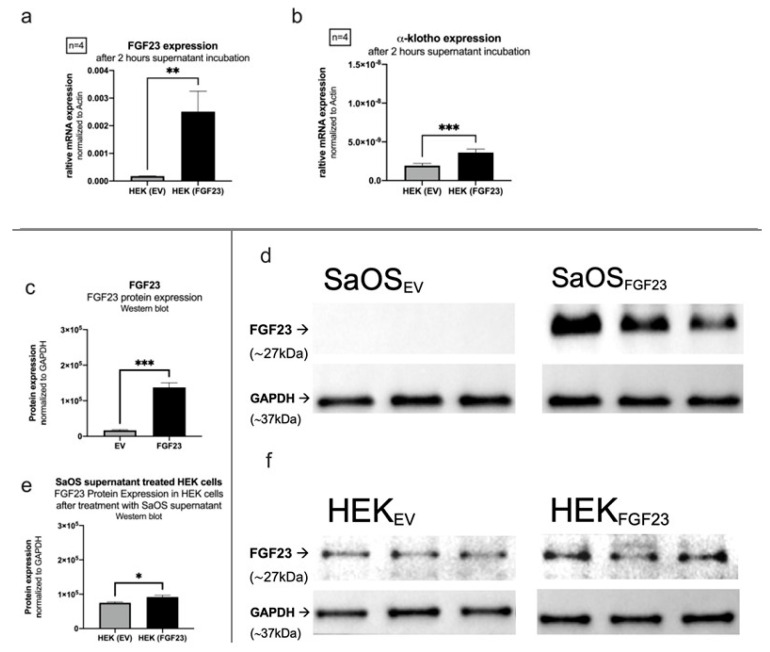
Detection of FGF23. HEK cells incubated with supernatant control or FGF23-overexpressing SaOS-2 cells. (**a**) FGF23 expression on mRNA level. (**b**) alpha-klotho expression on mRNA level. (**c**,**d**) FGF23 protein expression in SaOS-2 cells. (**e**,**f**) FGF23 protein expression in HEK cells treated with SaOS-2 supernatant. (* *p* < 0.05, ** *p* < 0.01, and *** *p* < 0.001). [(**c**–**f**): n = 3].

Overexpression of FGF23 in SaOS-2 cells was validated through qPCR experiments using supernatants from control and FGF23 overexpressing cells, as well as Western blots from SaOS-2 cells and HEK cells incubated with FGF23 and control supernatants (Figure 9). Following a two-hour incubation of HEK cells with either of the two supernatants, the presence of FGF23 mRNA (Figure 9a) and its cofactor alpha-Klotho (Figure 9b) was determined through real-time polymerase chain reaction (rtPCR). Compared to the relative expression of FGF23 normalized to Actin and GAPDH in cells treated with the supernatant of empty vector control cells (EV; 0.0001790), a significant increase was observed in HEK cells exposed to the supernatant of FGF23 overexpressing cells (FGF23; 0.002510; *p* = 0.0013). Alpha-Klotho exhibited a similar significance (*p* = 0.00006), with a mean of 1.915×10^−9^ in EV and 3.618 ×10^−9^ in the FGF23 supernatant-exposed cells.

Protein expressions (Figure 9) were tested via Western blot after protein purification from SaOS-2 and HEK cells following incubation with SaOS-2 supernatant.

In the initial experiment, SaOS-2 cells from both groups (EV and FGF23; Figure 9d) were purified, and the FGF23 protein concentration was determined by semiquantitative analysis of the western blot. Revealing protein concentrations of 16.661 for EV and 137.347 for the FGF23-overexpressing cell model indicate significant (*p* = 0.0009) higher expressions in the latter. In the subsequent experiment (Figure 9e), HEK cells were incubated with the SaOS-2 supernatants of EV or FGF23, followed by purification for Western blotting. Again significant (*p* = 0.0357) higher protein concentrations were detected in FGF23 (91.658 ± 10.964) compared to EV (74.927 ± 5.809).

The experiment provides evidence that SaOS-2 cells with FGF23 overexpression not only exhibit increased FGF23 expression but may also increase its secretion compared to the control group.

#### 3.2.3. Increased Stress and Apoptotic Signals upon FGF23 Overexpression

**Figure 10:**
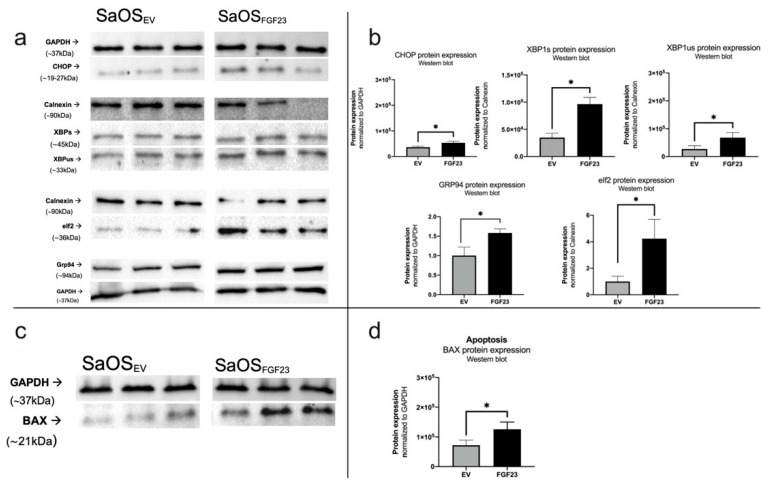
Western Blot ER-stress and apoptosis. Results of the Western Blots regarding the UPR at the protein level under the influence of FGF23 (**a**) and representation in bar diagrams with significance marked (**b**). The regulators of UPR were increasingly produced by FGF23. The UPR proteins are commonly upregulated by ER stress in the cell. (* *p* < 0.05). Results of the Western Blots regarding the determination of apoptosis under the influence of FGF23 (**c**) and representation in bar diagrams with significance marked (**d**). The apoptosis regulator BAX is found to be increased under the influence of FGF23. (* *p* < 0.05) [n = 3].

Western blots were conducted using proteins from various pathways, with a focus on ER stress (Figure 10a,b) and apoptosis (Figure 10c,d). Following protein purification of both control and FGF23-overexpressing SaOS-2 cells, alterations in the proteins of interest were analyzed and statistically validated using an unpaired *t*-test (Figure 10b). Notably, for CHOP, the mean intensity of the western blot bands after semiquantitative analysis was 36477 in control cells and 53,495 in FGF23-overexpressing cells, indicating a significant increase (SD: EV: 2452, FGF23: 3837; *p* = 0.0202). Similarly, XBP1spliced exhibited a substantial increase from 34,869 in control cells to 96,305 in FGF23-overexpressing cells (SD: EV: 7875, FGF23: 12,357; *p* = 0.0138). Additionally, the protein concentration of XBP unspliced was significantly lower in control cells (mean: 26,977) compared to cells influenced by FGF23 (mean: 67,904) (SD: EV: 7121, FGF23: 10,271; 0.0307). The mean expression of GRP94 was 1000 in control cells and 1583 in FGF23-overexpressing cells, indicating significance with *p* = 0.0136 (SD: EV: 0.1246, FGF23:0.06043). Moreover, the difference in eIf2 was also significant (*p* = 0.0205), with a mean of 1000 in control cells and 4233 in FGF23-overexpressing cells (SD: EV:0.2358, FGF23:0.8368). The apoptosis protein Bax exhibited upregulation as well (Figure 10c,d), with a mean of 72,145 in control cells and 125,265 in FGF23-overexpressing cells, significantly higher (SD: EV:9755, FGF23: 14,093; *p* = 0.0362).

The Western blot analysis reveals upregulated protein leves such as CHOP, XBP1 spliced, XBP1 unspliced, BAX, GRP94, and eIf2 in response to increased stress on the rER due to FGF23 overexpression. Consequently, cellular regulation appears to be initiated through various mechanisms, typically in response to rER stress. The elevated presence of the apoptosis protein BAX in cells influenced by FGF23 suggests an increased propensity for targeted cell death in these cells.

## 4. Discussion

Our study reveals significant alterations in SaOS-2 cells induced by FGF23. On the one hand, we found morphological differences in cellular organelles such as the rough endoplasmic reticulum (rER) and mitochondria, which are significantly enlarged and elongated in SaOS-2 cells overexpressing FGF23. In addition, the contact zones between these organelles are notably more pronounced, suggesting a closer interaction under the influence of FGF23. This morphological enlargement and increased organelle contact may indicate an adaptation to the increased protein synthesis and secretion demands imposed by FGF23 overexpression. Further molecular studies may indicate that not only elevated FGF23 expression but also secretion is associated with higher apoptosis rates after 24–72 h of culture, and upregulated protein levels are associated with ER stress and apoptosis. These findings suggest a mechanistic link between FGF23 signaling and cellular morphology, potentially implicating FGF23 in the regulation of cellular homeostasis and stress response pathways.

While the relationship between stress and other endocrine growth factors has been explored in the literature, FGF23 remains relatively unexplored. Previous research has linked ER stress to the regulation of FGF15, FGF19, and FGF21, with FGF21 also associated with mitochondrial stress [15]. However, the role of FGF23 in stress pathways has been largely overlooked, although it is known to be associated with inflammation, renal and cardiovascular disease, and proinflammatory pathways [23].

The morphological abnormalities of the rER and the mitochondria already indicate cellular stress. Underscored by the closer rER-mitochondrial contact zones, which in 2021, Kumar et al. have shown to be a sign of ER stress [17], as well as the higher apoptosis rate. The literature highlights the critical role of mitochondria-endoplasmic reticulum contact sites (MERCS) in regulating cellular homeostasis and responding to various stress conditions, including tumor microenvironmental stress and ischemic/hypoxic stress [24,25,26], but to date nothing is known about MERCS in the context of X-linked hypophosphatemia (XLH).

Another finding was the elevated FGF23 protein concentration and secretion. In a broader context, endocrine FGFs exert significant influence over various metabolic processes such as bile acid, glucose, fatty acid, phosphate, and vitamin D homeostasis [27]. Both FGF23 and ER stress have been implicated as contributing factors to conditions like overweight and obesity, which are often found in XLH [7]. ER stress, for instance, influences obesity by dampening energy expenditure, suggesting its potential as a therapeutic target for obesity treatment [28,29]. FGF23, on the other hand, shows positive associations with central and abdominal obesity [30,31]. Notably, the induction of ER stress by FGF23 may play a role in the severe obesity observed in patients with XLH, suggesting FGF23 as a potential trigger for metabolic dysregulation and increased fat accumulation.

Further protein analysis revealed significant increases in CHOP, XBP1 (both spliced and unspliced forms), GRP94, eIF2α, and BAX, indicating activation of the unfolded protein response (UPR) and subsequent apoptotic pathways. The protein CHOP (C/EBP homologous protein) is a key mediator of ER stress-induced apoptosis. Its role in promoting cell death in response to prolonged ER stress has been well documented [1]. Elevated CHOP levels in our study suggest that FGF23 overexpression induces a severe ER stress response leading to apoptosis.

XBP1 (X-box binding protein 1) exists in two forms: unspliced (XBP1u) and spliced (XBP1s). XBP1s is a potent transcription factor activated during the UPR to increase ER protein folding capacity and restore homeostasis [32]. The significant increase in both forms of XBP1 in our study indicates a robust UPR activation, perhaps aimed at coping with the increased protein load due to FGF23 overexpression.

GRP94 (glucose-regulated protein 94) is a chaperone involved in protein folding within the ER. Its upregulation is a hallmark of ER stress and functions to stabilize and correctly fold nascent proteins under stress conditions [33]. The increased GRP94 levels in our study indicate an attempt to cope with the increased protein synthesis demands induced by FGF23.

Phosphorylation of eIF2α (eukaryotic initiation factor 2 alpha) is a critical event in the UPR that leads to a reduction in global protein synthesis while selectively translating UPR-specific mRNAs, such as ATF4, to alleviate stress [34]. The elevated eIF2α levels in our results suggest an adaptive response to reduce the protein load entering the ER.

BAX (Bcl-2-associated X protein) is a long-known pro-apoptotic member of the Bcl-2 protein family [35]. Its upregulation triggers mitochondrial outer membrane permeabilization (MOMP), leading to cytochrome c release and activation of the apoptotic cascade [36]. The increased BAX expression in our study underscores the activation of apoptotic pathways due to prolonged ER stress and mitochondrial dysfunction.

To gain a more comprehensive understanding of the precise signaling cascade and the various pathways activated during ER stress, additional Western blots were attempted to provide a more exhaustive depiction of the protein response. Unfortunately, despite numerous attempts and adjustments to protocols, certain persistent challenges remained unresolved. Considerable efforts were made to modify protocols based on prior literature and manufacturer’s guidelines. However, difficulties were encountered with several antibodies targeting key proteins associated with ER stress, apoptosis, and mitochondrial dynamics, including phospho-IRE1alpha, ATF6, phospho-PERK, BiP/Grp78, Calreticulin/CALR3, XBP1, cleaved caspase 3, OMA1, and OPA1.

Nevertheless, these findings are consistent with the role of FGF23 in bone metabolism and its dysregulation leading to skeletal pathologies like X-linked hypophosphatemia (XLH). The observed ER stress and mitochondrial alterations can be interpreted as cellular responses to FGF23 overexpression, mirroring conditions seen in XLH where phosphate homeostasis is disrupted. The increased levels of UPR markers such as CHOP and GRP94 in our study highlight the cellular stress response, which is critical for understanding the pathophysiology of XLH and related disorders.

Moreover, the possible secretion of excess FGF23 from SaOS-2 cells suggests that overexpression not only affects intracellular pathways but also has paracrine effects, potentially influencing neighboring cells and contributing to the systemic nature of XLH. In future experiments, a direct measurement of FGF23 levels should be considered in addition to the indirect approach used in this study.

Future research should focus on elucidating the precise molecular mechanisms by which FGF23 induces ER stress and apoptosis, as well as exploring potential therapeutic targets to mitigate these effects. Understanding the broader implications of FGF23 overexpression on bone and systemic health could lead to improved treatments for skeletal diseases.

## 5. Conclusions

Our study reveals key insights into the cellular effects of FGF23 overexpression in SaOS-2 cells, highlighting a significant impact on organelle morphology, cellular stress responses, and apoptosis. We observed marked enlargement and elongation of the rough endoplasmic reticulum (rER) and mitochondria, alongside increased contact zones between these organelles, suggesting a closer interaction and adaptation to elevated protein synthesis demands. The upregulation of ER stress markers such as CHOP, XBP1, GRP94, and eIF2α, coupled with increased apoptosis rates, underscores the activation of the unfolded protein response (UPR) and the subsequent induction of apoptosis. These findings link FGF23 signalling, ER stress, and mitochondrial dysfunction, potentially contributing to the pathophysiology of X-linked hypophosphatemia (XLH).

Given the challenges encountered with certain molecular analyses, future research should aim to clarify the precise signaling pathways involved in FGF23-induced ER stress and apoptosis. Further investigation into the paracrine effects of FGF23 and its systemic implications could lead to novel therapeutic strategies for managing XLH and related skeletal disorders.

The study further implicates FGF23 in broader metabolic disturbances, possibly linking it to the obesity commonly observed in XLH patients. The evidence of ER stress and mitochondrial abnormalities provides a basis for exploring the systemic effects of FGF23 overexpression, which may extend beyond bone metabolism to affect overall cellular homeostasis and stress responses.

## Figures and Tables

**Table 1:** Western blotting antibodies.

Antibody	Source	Supplier	Dilution
FGF23	goat	abcam, Cambridge, UK, #ab56326	1:1000
GADD153/CHOP	rabbit	Novus USA, Centennial, Colorado, USA, #NBP2-13172	1:1000
eIf2 (D-3)	mouse	Santa Cruz Biotechnology, Dallas, TX, USA, #sc-133132	1:500
XBP1 (spliced/unspliced)	rabbit	Novus USA, Centennial, Colorado, USA	1:1000
Cleaved Caspase-3 mAB	rabbit	Cell signalling, Cambridge, UK #9664	1:1000
BAX	mouse	Santa Cruz Biotechnology, Dallas, TX, USA, #sc-20067	1:200
GAPDH	mouse	Santa Cruz Biotechnology, Dallas, TX, USA, #sc-365062	1:500
Calnexin	mouse	Santa Cruz Biotechnology, Dallas, TX, USA, #sc-23954	1:200
Anti-goat IgG (H+L) Secondary Antibody, HRP	donkey	Thermo Fisher Scientific, Waltham, Massachusetts, USA #A15999	1:10,000
Anti-mouse IgG (H+L)-HRP conjugate	goat	#1706516, Bio-Rad, Hercules, California, USA	1:10,000
anti-rabbit IgG (H+L)-HRP conjugate	goat	#1706515, Bio-Rad, Hercules, California, USA	1: 10,000

**Table 2:** Used real-time PCR primers.

Gene	Sequence (Human; 5′→3′)
Actin	AAACTGGAACGGTGAAGGTG CTCGGCCACATTGTGAACTTT
GAPDH	AACTTTGGTATCGTGGAAGG CAGTAGAGGCAGGGATGATGT
FGF23	GGAACAGCTACCACCTGCAGAT CACCACAAAGCCAGCATCCTCT
Alpha-Klotho	GATAGAGAAAAATGGCTTCCCTCC GGTCGGTAAACTGAGACAGAGTGG

## Data Availability

The raw data supporting the conclusions of this article will be made available by the authors on request.
